# Multi-Modal Biomarker Profiling of Tumor Microenvironment and Genomic Alterations to Enhance Immunotherapy Stratification in Melanoma

**DOI:** 10.3390/cimb47100821

**Published:** 2025-10-03

**Authors:** Meshack Bida, Thabiso Victor Miya, Tebogo Marutha, Rodney Hull, Mohammed Alaouna, Zodwa Dlamini

**Affiliations:** 1 The National Health Laboratory Service, Tshwane Academic Division, Department of Anatomical Pathology, University of Pretoria, Hatfield 0028, South Africa; 2 SAMRC Precision Oncology Research Unit (PORU), DSTI/NRF SARChI Chair in Precision Oncology and Cancer Prevention (POCP), Pan Africa Cancer Research Institute (PACRI), University of Pretoria, Hatfield 0028, South Africa; 3 Wolfson Wohl Cancer Research Centre, School of Cancer Sciences, University of Glasgow, Garscube Estate, Switchback Road, Bearsden, Glasgow G61 1QH, UK

**Keywords:** cutaneous melanoma, tumor mutational burden, tumor-infiltrating lymphocytes, cytokine signaling, immune escape, genomic profiling, immune checkpoint inhibitors, copy number variations

## Abstract

Tumor mutational burden (TMB) and tumor-infiltrating lymphocytes (TILs) are key biomarkers for predicting immunotherapy responses in cutaneous melanoma. The discordance between brisk TIL morphology and absent cytokine signals complicates immune profiling. We examined the interactions between TMB, TIL patterns, cytokine expression, and genomic alterations to uncover immune escape mechanisms and refine prognostic tools. A structure-based BRAF druggability analysis was performed to anchor the genomic findings in a therapeutic context. Primary cutaneous melanoma cases (N = 205) were classified as brisk (*n* = 65), non-brisk (*n* = 60), or absent TILs (*n* = 80) according to the American association for cancer research (AACR) guidelines. Inter-observer concordance was measured using intraclass correlation. Tumor necrosis factor alpha (TNF-α) and interferon gamma (IFN-γ) levels were graded using immunohistochemistry. Eleven brisk TIL cases lacking TNF-α expression were analyzed using the (Illumina TruSight Oncology 500, Illumina-San Diego, CA, USA). Dabrafenib docking to the BRAF ATP site was performed with Glide SP/XP and rescored with Prime MM-GBSA. Brisk TILs lacking cytokine signals suggested post-translational silencing of TNF-α/IFN-γ. Among the 11 profiled cases, eight exhibited high TMB and copy number alterations, with enrichment of nine metastasis/immune regulation genes. Inter-observer concordance was high (absent TILs, 95%; brisk TILs, 90.7%). BRAF docking yielded a canonical type-I pose and strong ATP pocket engagement (ΔG_bind −84.93 kcal·mol^−1^). Single biomarkers are insufficient for diagnosis. A multiparametric framework combining histology, cytokine immunohistochemistry (IHC), and genomic profiling enhances stratification and reveals immune escape pathways, with BRAF modeling providing a mechanistic anchor for the targeted therapy.

## 1. Introduction

Cutaneous melanoma is an aggressive skin cancer with a rising global incidence and considerable heterogeneity in its clinical behavior, treatment response, and prognosis. The advent of immune checkpoint inhibitors (ICIs) has significantly improved the outcomes of patients with advanced melanoma. However, the response to ICIs varies markedly among individuals, necessitating the identification of robust biomarkers to guide therapy. Among the most promising candidates are tumor mutational burden (TMB), tumor-infiltrating lymphocytes (TILs), cytokine expression signatures, and gene expression profiling (GEP). Individually, these biomarkers provide partial insights; however, combined, they may offer a more comprehensive strategy for predicting treatment response and disease progression. TMB, defined as the number of nonsynonymous mutations per megabase of the coding genome, has been proposed as a surrogate marker for neoantigen load and tumor immunogenicity [1]. Tumors with a high TMB are hypothesized to express more neoantigens, potentially enhancing their recognition and destruction by cytotoxic T cells. Studies such as the KEYNOTE-158 trial used a threshold of ≥10 mutations/Mb to stratify patients who are more likely to benefit from anti–PD-1 therapy [2]. However, the predictive value of TMB remains inconsistent across cancer types and even within melanoma. However, not all patients with high TMB respond favorably to ICIs, suggesting the influence of additional immunologic and molecular factors [3,4].

Tumor-infiltrating lymphocytes (TILs) are well-established prognostic factors in melanoma. A brisk TIL pattern, often observed in histopathological evaluations, is associated with improved survival and better immune response. However, some tumors classified as having brisk TILs exhibit weak or absent immunohistochemical (IHC) expression of key pro-inflammatory cytokines, such as tumor necrosis factor—alpha (TNF-α) and interferon-gamma (IFN-γ). This discrepancy likely reflects post-translational modification or suppression of cytokines within the tumor microenvironment (TME), impairing effective immune activation despite the morphological presence of TILs [5]. These findings underscore the limitations of relying solely on morphology and reinforce the need for molecular validation of immune activity. Copy number variations (CNVs) also contribute to the biology of melanoma. Gains in loci, such as 11q13 and 8q24 (harboring oncogenes such as *CCND1* and *MYC*), are associated with increased proliferation and metastatic potential, whereas deletions in regions such as 1p36 and 8p have been linked to poor prognosis [6,7]. Importantly, when CNVs are evaluated alongside TMB and mutation frequency, particularly in melanoma-associated genes, they enhance the specificity and sensitivity of prognostic models [6,8]. Gene expression profiling (GEP) has gained momentum as a clinical tool for further refining patient stratification. Commercial assays, such as the 31-GEP test (DecisionDx-Melanoma) and the 8-GEP test (MelaGenix), evaluate panels of metastasis-associated genes to predict recurrence risk and survival outcomes [9,10].

Lastly, while programmed death-ligand 1 (PD-L1) expression remains a U.S. Food and Drug Administration (FDA)-approved biomarker for predicting ICI response; however, its utility is limited by spatial and temporal heterogeneity within tumors [4,11]. Given these inconsistencies in the use of prognostic markers, this study aimed to investigate the association between tumor mutational burden and immune cell infiltration in cutaneous melanoma by integrating genomic, transcriptomic, and immunohistochemical data. Specifically, we sought to identify differentially expressed genes between the high- and low-TMB groups, assess functional pathways linked to immune and cytokine signaling, and explore how these features relate to histologic TIL patterns and cytokine expression to develop a more comprehensive framework for predicting immunotherapy response.

## 2. Materials and Methods

### 2.1. Sample Collection and Stratification

This retrospective cross-sectional study included 205 cases of primary early stage nodular and acral lentiginous cutaneous melanoma, retrieved from archival paraffin-embedded (FFPE) tissue blocks in a tertiary pathology service, the National Health Laboratory Services. All cases were diagnosed between 2020 and 2024 and selected to represent a range of TIL patterns. These melanoma cases were assessed as having a Breslow tumor thickness of 2.5 to 4 mm, defined as stage I and II AJCC with an adequate vertical growth phase. The clinical information was anonymized, and only relevant histopathological data were used.

Inclusion criteria

The cases were selected based on the following criteria:

- Early-stage histologic diagnosis of nodular cutaneous melanoma (Breslow tumor thickness of 2.5 to 4 mm), which corresponds to AJCC stage I (II).
- No surface ulceration.
- Whole-tumor representation in the tissue section.

Exclusion criteria:

- Incongruency in the histopathologic diagnosis of nodular cutaneous melanoma.
- Lack of full tumor depth representation in histologic tissue sections.
- Skin surface ulceration.
- Poor quality of histologic tissue section.

Hematoxylin and Eosin

(Automated Tissue-Tek Prisma, Sakura Finetek-Torrance, CA, USA) was used to prepare the tissue sections, while for immunohistochemistry, an Automated Benchmark XT machine supplied by (Roche, Basel, Switzerland) was used. An indirect method of 3.3 diaminobenzidine detection. The polyclonal antibody supplied by (SMM, Midrand, South Africa) and the polyclonal IFN-gamma antibody supplied by (Biocom Africa, Centurion, South Africa) were used.

### 2.2. Evaluation of Immune Cell Infiltration

TILs were morphologically assessed on hematoxylin and eosin-stained sections through independent examination by three senior pathologists and classified into brisk (65 samples), non-brisk (60 samples), or absent (80 samples) categories following the American Association for Cancer Research (AACR) guidelines. All samples underwent immunohistochemical (IHC) staining for TNF-α and IFN-γ using a (Tissue-Tek Prisma & Cover slipper HQplus stainer, Sakura Finetek-Torrance, CA, USA). The expression of these cytokines was scored semi-quantitatively as negative, weak, moderate, or strong, and was used to derive immune activation scores. IHC staining allowed for the evaluation of the functional immune status of the TME. Despite the brisk TIL morphology, discordant cases with absent IHC cytokine signals were identified.

#### 2.2.1. Histopathological Assessment of TILs

**Figure 1:**
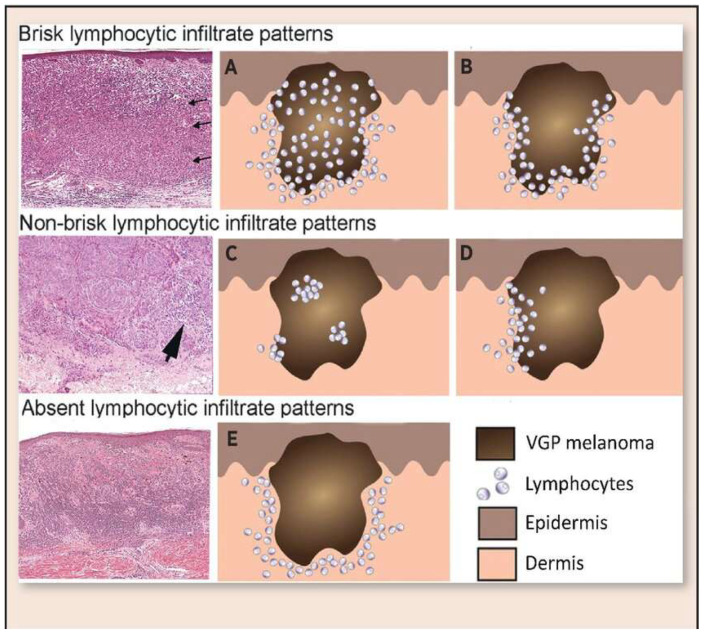
Morphological patterns of tumor-infiltrating lymphocytes (TILs) according to the AACR classification tool. (**A**) Brisk TIL histological section showing dense peri-tumoral lymphocytic infiltration closely applied around the tumor perimeter (arrows). (**B**) Brisk TIL schematic representation demonstrating peri-tumoral lymphocytes with variable intra-tumoral extension. (**C**) Non-brisk TIL histological section showing patchy or focal lymphocytic infiltration along parts of the tumor perimeter (arrowhead). (**D**) Non-brisk TIL schematic representation illustrating incomplete peri-tumoral coverage by lymphocytes. (**E**) Absent TIL histological section and schematic showing lack of appreciable lymphocytic infiltrate at the tumor–stroma interface.

Histopathological assessment of TILs was performed on hematoxylin and eosin (H&E)-stained sections. TILs were classified according to the American Association for Cancer Research (AACR) guidelines into three distinct categories (Figure 1): brisk, non-brisk, and absent. Brisk TILs were defined as continuous, dense lymphocytic infiltrate encompassing more than 50% of the tumor perimeter and/or diffusely infiltrating tumor cell nests. Non-brisk TILs were characterized by focal or patchy infiltrates involving less than 50% of the tumor perimeter or scattered infiltration among tumor cells. Absent TILs were determined by the near-complete lack of lymphocytic infiltration within or around tumor cell nests, excluding perivascular lymphocytes or those confined to the fibrous septae.

#### 2.2.2. TIL Scoring Procedure and Reproducibility Analysis

To ensure objectivity, three experienced anatomical pathologists independently scored all 205 melanoma cases without access to the molecular data. In instances where the two pathologists agreed on the TIL category, this consensus classification was adopted. In cases where all three pathologists provided differing scores, the median value was recorded as the final classification. The interobserver reproducibility of TIL scoring was assessed using the intraclass correlation coefficient (ICC) under a two-way mixed-effects model, where ICC values less than 0.5, 0.5–0.75, 0.75–0.9, and >0.9 indicated poor, moderate, good, and excellent agreement, respectively. All statistical analyses were performed using IBM SPSS software version 28.

#### 2.2.3. Reproducibility of TIL Scoring

The reproducibility of TIL scoring was analyzed across three experienced anatomical pathologists who independently evaluated all cases according to the AACR guidelines. Statistical assessment using the intraclass correlation coefficient (ICC) revealed excellent inter-observer agreement, particularly for categories at the extremes of the TIL spectrum. For cases classified as brisk TILs, the ICC was 0.90, indicating a high consistency. The absence of TIL cases demonstrated an even higher ICC of 0.95 (Table 1 and Table 2). However, moderate variability was noted in the intermediate category of non-brisk TILs, where differentiation between sparse and focally dense infiltrates was somewhat subjective. Nevertheless, the results underscore that when standardized criteria are rigorously applied, visual TIL scoring remains a highly reproducible technique in melanoma pathology.

### 2.3. Comprehensive Genomic Profiling

In the brisk TIL group, we selected 11 TNF-α-negative instances for further investigation. This involved comprehensive genomic profiling using the (Illumina TruSight Oncology 500 assay, Illumina-San Diego, CA, USA), allowing the analysis of tumor mutational burden (TMB), copy number variations (CNVs), single-nucleotide variants (SNVs), insertions and deletions (InDels), gene fusions, and splice variants. TMB was calculated as the number of non-synonymous somatic mutations per megabase. This comprehensive profiling provided insights into tumor genomic instability and neoantigen load, which are crucial for understanding immune responsiveness. The 11 TNF-α-negative cases were subjected to detailed analysis of CNVs and mutation frequencies [12].

### 2.4. Pathway Analysis and Target Gene Selection

We performed gene set enrichment analysis (GSEA) targeting metastasis-associated melanoma-related genes. Specifically, GSEA focused on immune escape pathways, particularly MAPK and PI3K/AKT signaling pathways. Genes such as MAPK1 and PIK3CA were selected based on their roles in regulating the immune response and tumor progression. TMB and gene expression profiles within these pathways were analyzed to further stratify the cytokine-negative cohort and elucidate the mechanisms underlying immune dysfunction [13].

### 2.5. Quality Control and Data Analysis

Sequencing data quality was rigorously assessed using standard metrics: sequencing depth, coverage uniformity, base quality, and mapping accuracy to the human reference genome. Samples that failed to meet the quality thresholds were excluded. Data processing and variant calling followed established bioinformatics pipelines that were optimized for cancer genomics research.

Among the TNF-α–negative cases, nine genes were significantly enriched; eight exhibited high TMB and CNVs, while three showed low levels of both.

### 2.6. BRAF–Dabrafenib Docking and MM-GBSA Rescoring (Schrödinger)

Dabrafenib is a selective ATP-competitive inhibitor of mutant BRAF kinase, particularly BRAF V600E, which targets the MAPK/ERK signaling pathway, a pathway that is hyperactivated in many cancers. It has been approved for the treatment of unresectable metastatic melanoma. It binds to the ATP-binding pocket of BRAF, stabilizing the inactive conformation [14]. We performed a structure-based analysis of BRAF to evaluate its druggability using Dabrafenib (Maestro version 14.5.131, MMshare Version 7.1.131, Release 2025-3, Platform Windows-x64), thereby linking our genomic findings to a potential therapeutic strategy.

*Target and ligand preparation*: The human BRAF kinase domain (ATP site) was prepared using Maestro with the Protein Preparation Wizard (OPLS4; pH 7.0). Missing side chains were rebuilt, bond orders/disulfides were assigned, hydrogens were added, and the structure was restrained and minimized. Dabrafenib was prepared with LigPrep/Epik (state enumeration at pH 7.0 ± 0.5; original stereochemistry retained).

*Receptor grid and docking*: A receptor grid was centered on the ATP/hinge region (Cys532/Leu514–Leu515 vicinity) and sized to encompass the hinge, P-loop, and DFG/activation segment pocket. The docking process was performed in two stages.

1. Glide SP was used to explore ligand conformations/placements with post-docking minimization.
2. Glide XP re-docking/refinement of the top SP poses as a cross-checking process.

The final pose was selected from the SP stage by consensus of the SP score and Emodel, along with canonical hinge recognition (dual H-bonds to Cys532) and the absence of clashes. XP results were reviewed for consistency but were not used for final ranking.

*MM-GBSA rescoring*: Prime MM-GBSA (VSGB 2.1; OPLS4) estimated the binding free energy (ΔG_bind) for the selected SP pose with side-chain relaxation for residues within 5 Å of the ligand. The reported value (see Table 3) for the chosen entry dabrafenib_LigPrep_pH7 was ΔG_bind = −84.93 kcal·mol^−1^.

*Interaction depiction*: A 3D pose view and 2D Ligand Interaction Diagram (LID) were exported from Maestro, summarizing the hydrogen bonds, hydrophobics, π-interactions, and polar contacts (default Schrödinger cutoffs). The LID serves as a static interaction fingerprint for the pose.

## 3. Results

### 3.1. Immune Infiltration Cytokine Immunohistochemistry

**Figure 2:**
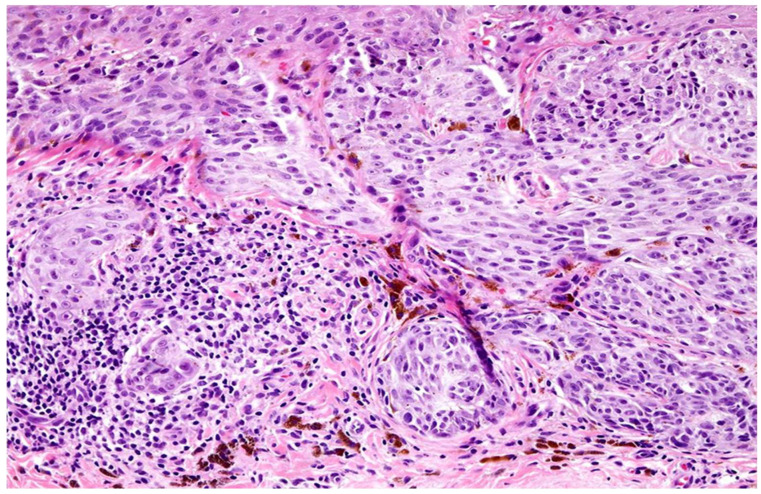
Melanoma tumor infiltrating lymphocytes, light micrograph. Tumor-infiltrating lymphocytes (TILs) in melanoma, showing a dense lymphocytic infiltrate within and around the melanoma, appear to correlate with a better prognosis; brisk TILs are present throughout the vertical growth phase or across. Original magnification ×20.

From an initial pool of 385 archived primary cutaneous melanoma cases diagnosed between 2020 and 2024, 205 samples were selected based on predefined inclusion and exclusion criteria. Tumor-infiltrating lymphocytes (TILs) were classified into brisk (*n* = 65), non-brisk (*n* = 60), and absent (*n* = 80) categories by three senior pathologists according to the AACR guidelines (Figure 2). Inter-rater reliability was assessed using intra-class correlation coefficients (ICC), yielding values of 0.90 and 0.95 for brisk and absent TILs, respectively, indicating excellent agreement. Discordant cases were resolved by averaging the scores from two pathologists in agreement (Table 1).

Among the brisk TIL cases, 11 samples exhibited absent TNF-α staining by immunohistochemistry (IHC), and six showed absent IFN-γ staining despite histologic evidence of immune infiltration. This discordance suggests potential post-translational modification or suppression of cytokine protein expression within the tumor microenvironment (TME).

### 3.2. Cytokine Immunohistochemical Expression in Melanomas with Brisk TILs

Immunohistochemical analysis was performed in all cases to assess the expression of TNF-α and IFN-γ as indicators of functional immune activity within the tumor microenvironment (Table 4). Among the 65 tumors classified as having brisk TILs, 11 (17%) exhibited a complete absence of TNF-α expression. Furthermore, six of these 11 TNF-α–negative cases also showed absent IFN-γ expression. This discordance between brisk TIL morphology and absent cytokine expression suggests the presence of morphologically prominent but functionally inert or suppressed TILs.

In contrast, most tumors with absent TILs demonstrated little to no cytokine expression, as anticipated, whereas the majority of brisk TIL cases exhibited moderate to strong TNF-α and IFN-γ staining. These observations highlight the potential limitations of relying solely on histologic TIL patterns to infer tumor immune activity.

#### 3.2.1. Cytokine Immunohistochemical Expression in Non-Brisk TIL Category

Among the non-brisk TIL cases, TNF-α expression was negative in seven cases (12%), weak in 19 (32%), moderate in 23 (38%), and strong in 11 (18%) (Table 4). IFN-γ expression was negative in eight cases (13%), weak in 24 cases (40%), moderate in 22 cases (37%), and strong in seven cases (12%). Compared with brisk TILs, the non-brisk cohort demonstrated an overall reduction in strong cytokine expression, reflecting a diminished immune response in these tumors.

#### 3.2.2. Cytokine Immunohistochemical Expression in Absent TIL Category

Interestingly, tumors histologically classified as having absent TILs occasionally exhibited substantial cytokine expression (Table 4). Among the absent TIL cohort, TNF-α expression was negative in 32 cases (40%), weak in 29 (36%), moderate in 13 (16%), and strong in six (8%). For IFN-γ, 39 (49%), 22 (28%), 15 (19%), and four (5%) cases were negative, weak, moderate, and strong, respectively. These results underscore the presence of latent immune activity in tumors that appear morphologically devoid of lymphocytic infiltrates. The absent TIL category corresponds to reduced cytokine transcriptomic genomic expression, as seen by the mRNA percentages lower than the expected range of 0 to 0.93 (i.e., <1), as shown in Table 5.

### 3.3. Mutational Analysis and Differential Gene Expression

**Figure 3:**
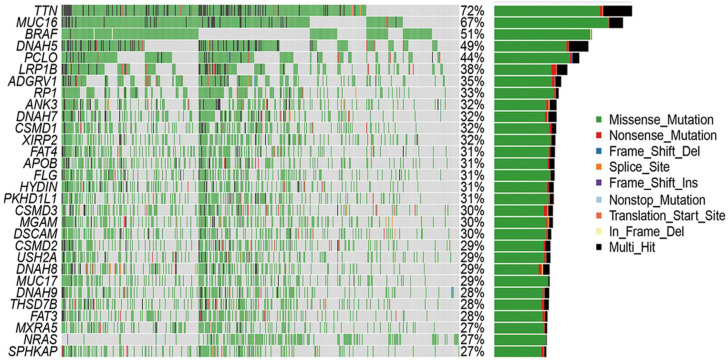
Analysis of somatic mutation profiles in TNF melanoma samples. A waterfall plot of detailed mutation information of the top 30 metastasis-associated genes with various color annotations to distinguish different mutation types, as well as to quantify TMB in percentage.

To investigate the underlying molecular mechanisms, gene expression profiling and genomic analyses were performed to elucidate the key signaling pathways driving tumor behavior in the cohort. The primary pathways implicated include MAPK, PI3K/AKT, and wingless-related integration site (WNT), with a particular focus on MAPK signaling (RAS/RAF/MEK/ERK) due to its established role in melanoma pathogenesis. Differentially expressed genes (DEGs) between the high- and low-TMB groups were identified using the FDA-approved KEYNOTE-158 cutoff of ≥10 mutations per megabase. A waterfall plot (Figure 3) of the top metastasis-associated differentially expressed genes (DEGs) revealed clear stratification by copy number variation (CNV) and tumor mutational burden (TMB). Pathway enrichment analysis implicated MAPK, PI3K/AKT, and WNT signaling pathways, with MAPK1 and PIK3CA showing elevated expression in TNF-α–negative cases. The top nine DEGs distinguishing these groups were identified and visualized in a heatmap, showing clear stratification linked to the TMB status (Figure 3).

Of the 11 TNF-α–negative samples, eight exhibited high TMB and significant CNVs, and three had low TMB and CNVs. Mutation profiling revealed frequent alterations in *TTN* (72%), MUC16 (67%), BRAF (51%), and other melanoma-associated genes. Missense mutations and SNPs, particularly C>T transitions consistent with UV-induced damage, were the most predominant. To further investigate immune escape pathways, expression profiles, and CNVs of key genes in TNF signaling and related pathways were analyzed, specifically focusing on *MAPK1* and *PIK3CA* genes within the 11 TNF-α negative IHC cases (Table 6). The arbitrary threshold for significant CNVs in melanoma was set at 18.1, consistent with the previous literature [7].

Notably, eight of the 11 TNF-α-negative cases exhibited a high mutational burden (≥10 mutations/Mb) alongside increased expression levels of MAPK1 and PIK3CA, despite the absence of cytokine protein detection. This finding supports the hypothesis that post-translational cytokine modification inhibits protein detectability in IHC despite active gene transcription and pathway stimulation.

### 3.4. Tumor Mutational Burden (TMB) and Mutation Frequency

**Figure 4:**
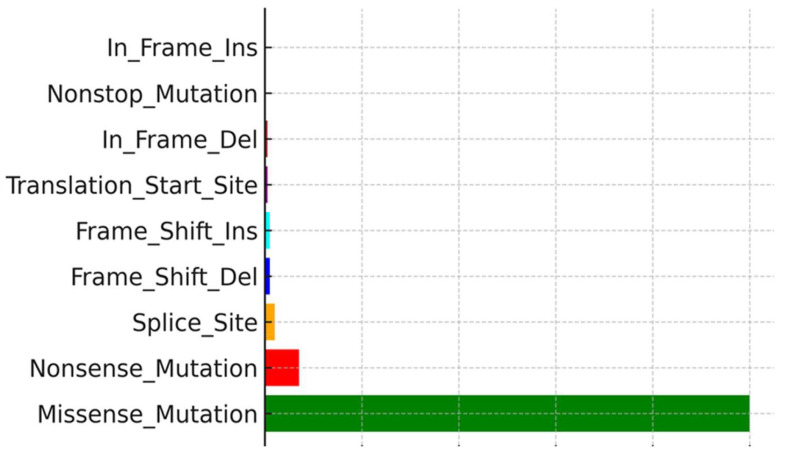
Variant classification. According to further comparison, missense mutations, SNPs, and C>T mutations accounted for the vast majority of the different classification categories.

**Figure 5:**
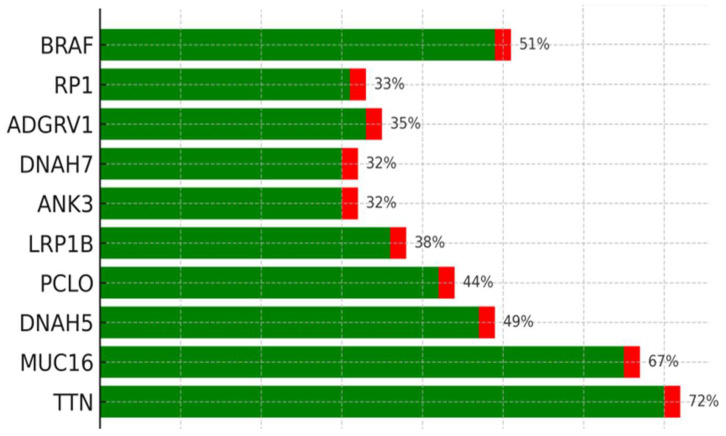
TMB in the top nine genes analyzed showed high mutation frequencies (Green = Missense Mutation, Red = Nonsense_Mutation). The genes identified were *TTN*, *MUC16*, *DNAH5*, *LRP1B*, *ANK3*, *DNAH7*, *ADGRV1*, and *RP1*.

Of the 11 TNF-α-negative cases, eight had a TMB, while three had a low TMB. Somatic mutation profiling was expanded to include TNF-α and IFN-γ IHC-negative patients, with 11 and six samples, respectively, to explore the mutation landscape associated with immune evasion. Among these samples, *TTN* (72%), *MUC16* (67%), *BRAF* (51%), *DNAH5* (49%), *PCLO* (44%), *LRP1B* (38%), *ADGRV1* (35%), *RP1* (33%), and *ANK3* (32%) were the most commonly mutated genes. Mutation type analysis revealed that missense variants constituted the majority of these mutations. Supporting visualizations include a waterfall plot showing somatic mutation profiles and frequencies (Figure 3), a variant classification showing the prevalence of missense mutations and SNPs, particularly frequent C>T transitions (Figure 4), and a plot showing the distribution of TMB across the top nine mutated genes (Figure 5).

#### 31-GEP Test

A 31-GEP for melanoma, often referred to as the “DecisionDx-Melanoma” test, analyses a panel of 27 prognostic genes alongside 3 control genes, including genes like *LGALS1*, *SULF1*, *COL4A1*, *ITGB3*, *PLAT*, *SERPINE2*, *GDF15*, *TGFBR1*, *LOXL4*, *CXCL8*, and *MLANA*. Although the exact list of 27 genes may vary depending on the specific 31-GEP test, they typically include genes associated with cell growth, invasion, and metastasis in melanoma [15].

The test generates a continuous score between 0 and 1, which is then categorized into four classes (1A, 1B, 2A, and 2B), representing an increasing risk of recurrence [16].

In most cases, the score correlated with the actual number of high-risk genes as well as the variant gene data metric, which include CNV, TMB as well as frequency of mutations.

Interpretation:

- Class 1A (Low risk): Indicates a low probability of melanoma recurrence.
- Class 1B/2A (Intermediate risk): Represents a moderate risk of recurrence.
- Class 2B (high risk): High likelihood of melanoma recurrence or metastasis.

An automated 31-gene expression melanoma result image is either reflected in color-coded risk levels, where the image usually uses colors to visually represent the risk categories, with green signifying low risk (Class 1) and red signifying high risk (Class 2). The 31-GEP test uses a color-coded system to classify melanoma risk levels into three categories: Class 1A (blue), Class 1B/2A (orange or red), and Class 2B (teal or red). Class 1A represents the lowest risk, Class 1B/2A represents intermediate risk, and Class 2B represents the highest risk group. 

A bar graph representation was extracted from a tabular variant gene data metric (expression levels based on mRNA copies; CNV and TMB can also be performed, which were used in this study as well, including bases of selected gene alteration data such as expression levels [17].

The 31-GEP is a useful tool for stratifying patients with cutaneous melanoma according to their risk of recurrence-free survival, distant metastasis-free survival, and melanoma-specific survival. It remains a significant and independent predictor of metastatic recurrence [18].

This 31-GEP test identifies high-risk patients who are likely to experience recurrence or die of melanoma within the low-risk groups of subpopulations of patients with CM who have SLN-negative disease, stage I to IIA tumors, and thin tumors.

Six TNF-alpha and four IFN-gamma absent TIL category samples were selected to determine the clinical significance of strong immunohistochemical signals by the 31-GEP test and demonstrated that three of six TNF-alpha and one out of four IFN-gamma absent TIL category samples were Class 1A, which means a low 5-year risk of recurrence or metastasis. This translates into 50% and 25% possible good immune responses in immunotherapy decisions, even though the numbers are low for a statistically significant finding. Further research with adequate sample sizes is required to validate these findings.

By identifying genomic biomarkers of high-risk metastatic potential using gene expression profiles, this study determined the clinical significance of strong immune scores in what is otherwise a poor TIL infiltrate in the tumor (Table 7). Using a computational method to explore the genomic data of RNA-seq based on the mRNA transcripts of these genes, the metastatic progression of these samples can be predicted and stratified according to their respective classes. The melanoma genome project provides a list of such genes, which are also included in TCGA. The metastatic progression scores from the RNA-Seq data can then be presented as computational colored heat maps [19].

The 31-GEP test from poor TIL infiltrates showed a favorable class 2B in some cases because of the rate of gene expression, which compensates for poor immune effector infiltration.

### 3.5. Mutation Type and Genomic Variation

Analysis of mutation types revealed SNPs, particularly C>T transitions, as the most common mutation type in these melanoma samples, consistent with UV-induced DNA damage signatures. The combined evidence of high TMB, specific mutational patterns, and lack of cytokine protein despite brisk TIL presence underscores the complexity of the immune escape mechanisms in cutaneous melanoma.

Overall, these findings show a major mismatch between brisk TIL histology and the absence of cytokine protein expression in some melanoma patients, which is most likely due to post-translational alterations of cytokines. A high TMB was observed in most TNF-α IHC-negative brisk TIL instances. However, TMB alone cannot predict successful immune activation in the absence of cytokine proteins. Mutation frequency analysis also revealed recurrent abnormalities in genes such as *TTN*, *MUC16*, and *BRAF*, which correspond to their recognized functions in melanoma development. Finally, an integrated analysis of TMB, CNVs, gene expression profiles, and immune cytokine states provides a more complete understanding of the mechanisms of immune evasion and possible predictors of immunotherapy response.

### 3.6. BRAF–Dabrafenib Docking and MM-GBSA

The Glide SP stage produced a pose that satisfied canonical hinge recognition and showed no steric clashes; Glide XP re-docking recovered the same orientation but was used only as a cross-check and not for the final ranking. The selected SP pose (Figure 1A,B) displayed dual hinge hydrogen bonds with Cys532, fixing the aminopyrimidine core. Around this anchor, a lipophilic shelf formed by Thr529, Leu514, Leu515, and Phe516 packs the hinge-proximal rings, whereas the P-loop (Gly464–Ser465) folds over the difluorophenyl group to provide a hydrophobic cap. Distally, Phe583 and the DFG motif (Asp594–Phe595) furnish π–π/edge-to-face packing, which stabilizes the tail of the scaffold. Lys483 engages the sulfonamide/heteroatom region Via polar interactions, and additional nonpolar residues (Ala481, Val471, Ile463, Ile527, and Ile592) form a tight hydrophobic cage. Gln530, Trp531, Asn580–Asn581, and Gly593 were proximal without explicit hydrogen bonds in this pose. This static interaction fingerprint matches the expected pharmacophore for active-like (DFG-in) BRAF inhibitors.

**Figure 6:**
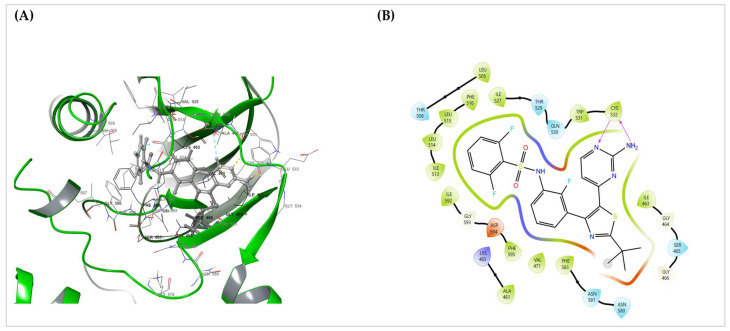
BRAF–dabrafenib binding mode and interaction map. (**A**) 3D pose of dabrafenib in the BRAF ATP site showing canonical type-I engagement: the aminopyrimidine core forms dual hinge hydrogen bonds with Cys532, whereas Thr529, Leu514, Leu515, and Phe516 create a lipophilic shelf around the hinge. The P-loop (Gly464–Ser465) caps the difluoro-phenyl ring, and distal stabilization arises from Phe583 and the DFG motif (Asp594–Phe595) Via π–π/edge-to-face packing interactions. Lys483 provides a polar clamp near the sulfonamide/heteroatom region, with additional nonpolar contacts from Ala481, Val471, Ile463, Ile527, and Ile592, completing a tight hydrophobic cage. Gln530, Trp531, Asn580–Asn581, and Gly593 are proximal without explicit hydrogen bonds. (**B**) The corresponding 2D Ligand Interaction Diagram summarizes this static interaction fingerprint (hinge dual H-bonds, P-loop capping, DFG/activation-segment packing, and β3-lysine polar contact), consistent with the favorable Prime MM-GBSA estimate for the selected Glide-SP pose (ΔG_bind = −84.93 kcal·mol^−1^).

Prime MM-GBSA rescoring of the chosen SP pose (dabrafenib_LigPrep_pH7) yielded ΔG_bind = −84.93 kcal·mol^−1^, indicating strong binding for this configuration (Table 3). Together with the contact map in (Figure 6A,B), these data provide a clear, mechanism-level anchor for the translational relevance of BRAF targeting in this cohort (Section 2.6).

Pose-quality checks further supported the chemical plausibility of the selected SP pose: the hinge H-bond geometry was ideal, no steric clashes or unsatisfied buried polar atoms were detected, and the fit exhibited good shape complementarity without requiring crystallographic water. XP re-docking reproduced the same binding orientation, indicating the robustness of docking. The contact pattern spans the adenine pocket (hinge), hydrophobic subpocket I at the gatekeeper/P-loop shelf, and subpocket II near the DFG/activation segment, which is consistent with a DFG-in/type-I inhibitor profile.

## 4. Discussion

In this study, we investigated the correlation between TMB and TILs in primary cutaneous melanoma using TCGA data. We analyzed DEGs between the high- and low-TMB groups to explore the immune genomic landscape and understand the implications of discordant IHC signals in cases exhibiting brisk TIL histomorphology but weak or absent TNF-α and IFN-γ expression. The utility of TIL as a prognostic biomarker in clinical practice is sometimes limited by subjectivity [20,21].

Despite this perceived subjectivity, many cases remain in which the concordance and fidelity of evaluation are acceptable, as observed in our study with excellent reliability (greater than 0.9 ICC values). The intraclass correlation coefficient (ICC) is a widely used reliability index in test–retest, interrater, and interrater reliability analyses [22].

Both TMB and TILs have been independently associated with improved response to ICIs in various malignancies; however, each marker has limitations when used alone. TMB quantifies the total number of nonsynonymous mutations per megabase and is associated with neoantigen production which can stimulate anti-tumor immune responses [1,3]. However, inconsistencies in treatment outcomes among patients with high TMB have raised concerns regarding its predictive accuracy [4]. Similarly, histological evidence of brisk TILs does not always correspond to functional immune activation, particularly when cytokine signaling is dysregulated in the TME [23].

Our findings align with those of prior studies suggesting that TILs in melanoma, although morphologically brisk, may be functionally compromised when key cytokines such as TNF-α and IFN-γ are absent at the protein level [23]. This likely reflects post-translational modification or suppression of cytokine expression in the TME of the tumor. Such immune evasion mechanisms may help explain the poor responses to ICIs in some high-TMB tumors, a finding consistent with reports from the KEYNOTE-158 study, which used a TMB threshold of ≥10 mutations/Mb to stratify responders to pembrolizumab [2]. This highlights the need to combine TMB with other biomarkers for better predictive accuracy. Several studies have reported that a high TMB contributes to a rich neoantigen landscape, potentially leading to enhanced immune infiltration and better immunotherapy outcomes [24,25]. However, high-TMB tumors may remain unresponsive if the critical cytokine signaling pathways are disrupted [26].

Our study reinforces the emerging consensus that TMB should be integrated with TILs, cytokine activity, and other immune and genetic markers to improve the prediction of ICI efficacy [4,11].

Although TMB and TILs have been extensively studied in colorectal cancer, data on melanoma are limited [6,27]. Our results demonstrate that cutaneous melanoma may exhibit high TMB and morphologically brisk TILs without corresponding cytokine signals, emphasizing the importance of a multidimensional immune profiling approach [28]. We used TruSight Oncology 500 (TSO 500) for comprehensive genomic profiling, detecting SNVs, InDels, CNVs, gene fusions, and splice variants, and precise TMB calculation. Nine highly mutated and proliferative genes were identified, aligning with previous reports of up to 28 genes associated with metastasis and progression in melanoma [9]. These genes may represent critical components of a gene expression signature that enhances risk stratification.

The growing use of GEP tests in melanoma management reflects the clinical demand for validated prognostic tools in melanoma. The 31-GEP test (Castle Biosciences), supported by the U.S. Medicare for select T1 and T2 melanomas identifies low- and high-risk patients using a panel of 31 genes [17]. Similarly, the 8-GEP test (MelaGenix), recently developed in Germany, stratifies Stage II melanomas into high- and intermediate-risk categories [10]. Our findings support the integration of GEP results with genomic and immunologic biomarkers for optimal clinical utility. PD-L1 expression remains the most accessible ICI biomarker, evaluated using IHC on the Dako Omnis platform with the 28–8 pharmDx antibody. However, its predictive reliability is limited by tumor heterogeneity and temporal variations [4,19]. The recognition of TIL as potential biomarkers of predictive prognosis within melanomas has been mooted by Hillen et al. [29]. Our data support the combination of PD-L1 expression with TMB, TIL evaluation, cytokine IHC, and genetic profiling for a more accurate predictive framework. The integration of genetic analysis into treatment decision-making processes is long overdue and will improve efficiency in the field of personalized medicine [30,31].

To translate the immunogenomic observations into a therapy-focused mechanism, we complemented the TCGA/TSO 500 findings with a structure-based assessment of BRAF-druggability. This is particularly relevant for cases in which high TMB and morphologically brisk TILs coexist with reduced TNF-α/IFN-γ protein levels, limiting the predictive value of single immune markers. Docking analysis provides a concrete molecular context for considering a targeted option when the ATP site architecture is preserved.

Docking of dabrafenib into the BRAF ATP site produced a chemically coherent Glide SP–selected pose (XP re-docking served as a cross-check). The pose reproduces hallmark type-I (DFG-in) recognition: dual hinge hydrogen bonds to Cys532; a gatekeeper/hinge shelf (Thr529, Leu514–Leu515, Phe516) that packs the hinge-proximal rings; P-loop capping (Gly464–Ser465) over the difluoro phenyl group; distal π–π/edge-to-face packing by Phe583 and the DFG motif (Asp594–Phe595); a Lys483 polar clamp; and a surrounding hydrophobic cage (Ala481, Val471, Ile463, Ile527, Ile592), with Gln530, Trp531, Asn580–Asn581, and Gly593 proximal without explicit hydrogen bonds (Figure 6).

The scoring summary (Table 3) indicates that the Glide SP pose used for analysis (dabrafenib_LigPrep_pH7-SP) had a Glide Score of −11.495, docking score of −11.090, and Emodel of −115.824, with a modest Epik ionization penalty of 0.4058, consistent with a physiologically plausible ligand state. XP re-docking preserved the same orientation but returned a less favorable XP GScore of −9.053 (docking score −8.648) despite a slightly more negative Emodel −117.95; accordingly, the SP pose was retained by the pre-specified SP score/Emodel criterion. Prime MM-GBSA on the same SP pose yielded ΔG_bind = −84.93 kcal·mol^−1^, and the minimized complex Prime Energy was −21,746.96, consistent with the strong engagement of the ATP-site. Together, the contact map and numerical scores/energetics support a coherent type-I binding mode for dabrafenib in the context of BRAF mutation in this cohort and illustrate how a targeted mechanism can be considered alongside TMB, standardized TIL assessment, cytokine IHC, PD-L1, and genomic profiling (Figure 6A,B and Table 3).

## 5. Conclusions

Identifying reliable predictive biomarkers for cutaneous melanoma remains challenging. Visual morphological TIL assessment using routine H&E staining is a practical, low-cost tool. We recommend scoring lymphoid aggregates along the entire stromal–tumor interface to improve concordance.

Single markers (TMB, TILs, PD-L1) are insufficient. A multi-step framework combining cytokine IHC, standardized TIL histology, and comprehensive genomic/transcriptomic profiling is preferable for ICI decision-making. Finally, structure-based modeling confirmed canonical type-I binding of dabrafenib to BRAF with favorable Glide SP scores and Prime MM-GBSA (ΔG_bind = −84.93 kcal·mol^−1^), supporting the consideration of BRAF-directed therapy within this composite approach.

## Figures and Tables

**Table 1:** ICC values with confidence intervals for brisk, non-brisk, and absent TIL categories.

Intraclass Correlation Coefficient							
	Intraclass Correlation ᵇ	95% Confidence Interval		F Test with True Value 0			
		Lower Bound	Upper Bound	Value	df1	df2	Sig
Single Measures	0.880 ᵃ	0.860	0.898	52.124	299	1794	0.000
Average Measures	0.981	0.977	0.984	52.124	299	1794	0.000

Two-way random effects model where both people effects and measures effects are random. ᵃ The estimator is the same whether the interaction effect is present or not. ᵇ Type C intraclass correlation coefficients using the consistency definition. The between-measure variance was excluded from the denominator variance.

**Table 2:** Inter-observer reliability of TIL classification, showing intra-class correlation coefficients (ICC) and corresponding 95% confidence intervals for brisk, non-brisk, and absent categories (*n* = 205).

Total = 205 Upper Bound (Brisk) = 65 Lower Bound (Absent) = 80	Intra-Class Correlation b	95% Confidence Interval	
		Lower Bound	Upper Bound
Single measures	180	76	59
Average measures	15	4	6
ICC value		0.95	0.9

**Table 3:** Prime MM-GBSA binding energy (ΔG_bind) for the selected BRAF–dabrafenib Glide-SP pose and key contacting residues.

Title	Ionization Penalty	Docking Score	Glide Emodel	Glide GScore	XP GScore	MMGBSA dG Bind	Prime Energy
BRAF_6V2U_prepared		-	-	-	-	-	-
dabrafenib_LigPrep_pH7-SP	0.4058	−11.09	−115.824	−11.495	-	-	-
dabrafenib_LigPrep_pH7-XP	0.4058	−8.648	−117.95	−9.053	−9.053	-	-
dabrafenib_LigPrep_pH7-prime_mmgbsa	0.4058	−11.09	−115.824	−11.495	-	−84.93	−21,746.96

**Table 4:** Ratio analysis for all TIL’s classification.

	Ratio Analysis of Expression Levels in Each TIL Category								
		TNF-α				IFN-γ			
	Total	Negative	Weak	Moderate	Strong	Negative	Weak	Moderate	Strong
Brisk	65	0.17	0.17	0.31	0.35	0.09	0.18	0.37	0.35
Non-brisk	60	0.12	0.32	0.38	0.18	0.13	0.40	0.37	0.12
Absent	80	0.40	0.36	0.16	0.08	0.49	0.28	0.19	0.05

**Table 5:** Overall QC metrics and transcriptomic TNF-alpha gene expression profile in the six cases of absent TIL category, showing a good quality yield.

Absent TIL × 6	Run Name	Run	PCT_PF_READS (80%)	DNA Median Exon Coverage (150) and %50×	DNA% Target Reads (250×/100×/50×) Median Target Coverage	RNA Reads	RNA% on Target Reads. Median Cov Gene 500× (0.00–0.93)	CNV Median Bin (>1)	TMB (12.4)	MSI (40)
TNF-Alpha										
OS/20-03227	2024-07-24_Mel06	Run2 sample13	Pass (88.8)	Pass (199) %Exon 50× = 99.6%	Pass (34.3/1.1/96.2) (MTC 206)	Pass ~22.8 mil	Pass (93.2) (MCG: 0.69)	Pass (5.6)	Pass (23)	Pass (54)
OS/21-21720	2024-07-24_Mel08	Run2 sample14	Pass (86.4)	Pass (194) %Exon 50× = 96.4%	Pass (31.6/72.0/94.8) (MTC 202)	Pass ~20.0 mil	Pass (89.9) (MCG: 0.63)	Pass (2.1)	Pass (15.6)	Pass (123)
OS/19-07423	2024-07-24_Mel13	Run2 sample15	Pass (84.2)	Pass (192) %Exon 50× = 95.6%	Pass (32.0/72.8/95.1) (MTC 203)	Pass ~20.7 mil	Pass (93.3) (MCG: 0.84)	Pass (1.8)	Pass (16.2)	Pass (64)
OS/20-03227	2024-07-24_Mel06	Run2 sample13	Pass (87.1)	Pass (199) %Exon 50× = 98.6%	Pass (31.8/72.1/94.9) (MTC 202)	Pass ~20.6 mil	Pass (93.2) (MCG: 0.69)	Pass (5.6)	Pass (20.3)	Pass (59)

**Table 6:** Differential expression and CNV data of MAPK1 and PIK3CA genes in TNF-α negative brisk TIL cases.

Gene Expression Level and Mutational Analysis in MAPK1 and PIK3CA Genes, Respectively										
							TMB		Expression Level	
Brisk TIL Categories	Immune Scores	DNA Data Coverage and Reads (80%)	CNV (18.1)	TMB (10 mut/Mb)	Freq/mut (20)	Genes Involved	Low	High	Low	High
Case 1	negative	80%	20.3; 18.5	13.2; 18.1	21.2; 22.4	MAPK1; PIK3CA		X		X
Case 2	negative	79.80%	22.6; 27.9	15.6; 13.9	23.5; 25.9	MAPK1; PIK3CA		X		X
Case 3	negative	82%	14.6;13.1	9.3; 8.8	7.2; 7.1	MAPK1; PIK3CA	X		X	X
Case 4	negative	84%	18.9; 20.4	16.7; 14.2	23.2; 19.1	MAPK1; PIK3CA		X		X
Case 5	negative	78.90%	19.5; 21.4	15.2; 16.5	23.4; 27.1	MAPK1; PIK3CA		X		X
Case 6	negative	86%	30.9; 24.1	19.1; 18.8	24.1; 24.4	MAPK1; PIK3CA		X		X
Case 7	negative	78.9%	12.9; 14.8	8.8; 9.1	15.3; 12.9	MAPK1; PIK3CA	X		X	
Case 8	negative	80.2%	27.1; 26.5	15.6; 16.8	26.2; 23.4	MAPK1; PIK3CA		X		X
Case 9	negative	80.4%	13.6; 12.8	7.9; 8.1	12.6; 13.5	MAPK1; PIK3CA	X		X	
Case 10	negative	80.6%	29.7; 28.4	16.9; 18.4	26.8; 24.7	MAPK1; PIK3CA		X		X
Case 11	negative	80.5%	30.4; 26.4	21.6; 17.9	29.1; 24.2	MAPK1; PIK3CA		X		X

The “X” symbolizes the TMB and Expression levels whether its Low/High.

**Table 7:** Gene expression profiling—TNFalpha in TIL absent category with strong immunohistochemical signal.

31-GEP Gene Signatures Expression Analysis—TNF-Alpha									
Gene Variant Data Expression Levels Rison—Using Baseline Data [*CDK1*; *FOXM1*; *KIF11*; *RFC4*; *CDK4*; *KIT*; *CDK2*]							Low Risk	Intermediate Risk	High Risk
Absent TIL categories	Immune scores	DNA data coverage and reads (80%)	CNV (18.1)	TMB (12.4 mut/Mb)	Freq/mut (20)	Genes involved	Class1A	Class 1B/2A	Class2B
Case 1	strong	80%	20.3	14.2	17.2	*CDK1*	1A		
Case 2	strong	79.80%	25.6; 24.9; 21.6	12.6; 14.9; 11.9	16.5; 14.9; 20.9	*CDK1*; *FOXM1*; *RFC4*			2B
Case 3	strong	82%	19.6; 20.1	19.3; 21.3	29.2; 25.1;	*KIF11*; *RFC4*		1B/2A	
Case 4	strong	84%	18.4; 17.4; 16.9; 20.4	12.7; 16.2; 13.4; 17.1	17.5; 23.1; 24.1; 26.4	*CDK2*; *CDK4*; *KIT*; *RFC4*			2B
Case 5	strong	78.90%	17.5; 23.4	14.2; 13.5	19.4; 20	*VWF*; *RFC4*		1B/2A	
Case 6	strong	86%	30.4; 26.1; 25.9; 27.4	12.1; 16.8; 12.4; 15.3	29.1; 26.4; 16.5; 24.1; 21.9	*RFC4*; *FOXM1*; *KIF11*; *KIT*; *VWF*			2B

## Data Availability

The original contributions presented in this study are included in the article. Further inquiries can be directed to the corresponding authors.

## Abbreviations

The following abbreviations are used in this manuscript:

AACR	American Association for Cancer Research
CNV	Copy number variation
FDA	U.S. Food and Drug Administration
FFPE	Formalin-fixed, paraffin-embedded
GEP	Gene expression profiling
GSEA	Gene set enrichment analysis
ICC	Intra-class correlation coefficients
ICIs	Immune checkpoint inhibitors
IFN-γ	Interferon-gamma
IHC	Immunohistochemical
InDels	Insertions and deletions
PD-L1	Programmed death-ligand 1
SNVs	Single-nucleotide variants
TILs	Tumor-infiltrating lymphocytes
TMB	Tumor mutational burden
TNF-α	Tumor necrosis factor—alpha
WNT	Wingless-related integration site
